# Comparing the Effectiveness of Education Versus Digital Cognitive Behavioral Therapy for Adults With Sickle Cell Disease: Protocol for the Cognitive Behavioral Therapy and Real-time Pain Management Intervention for Sickle Cell via Mobile Applications (CaRISMA) Study

**DOI:** 10.2196/29014

**Published:** 2021-05-14

**Authors:** Sherif M Badawy, Kaleab Z Abebe, Charlotte A Reichman, Grace Checo, Megan E Hamm, Jennifer Stinson, Chitra Lalloo, Patrick Carroll, Santosh L Saraf, Victor R Gordeuk, Payal Desai, Nirmish Shah, Darla Liles, Cassandra Trimnell, Charles R Jonassaint

**Affiliations:** 1 Department of Pediatrics Northwestern University Feinberg School of Medicine Chicago, IL United States; 2 Division of Hematology, Oncology and Stem Cell Ann & Robert H Lurie Children's Hospital of Chicago Chicago, IL United States; 3 Department of Medicine University of Pittsburgh Pittsburgh, PA United States; 4 Child Health Evaluative Sciences, Hospital for Sick Children Lawrence S Bloomberg Faculty of Nursing University of Toronto Toronto, ON Canada; 5 Child Health Evaluation Sciences Hospital for Sick Children Toronto, ON Canada; 6 Institute for Health Policy, Management & Evaluation University of Toronto Toronto, ON Canada; 7 Johns Hopkins Sickle Cell Center for Adults Department of Psychiatry and Behavioral Sciences Johns Hopkins School of Medicine Baltimore, MD United States; 8 Sickle Cell Center, Department of Medicine University of Illinois at Chicago Chicago, IL United States; 9 Ohio State Adult Sickle Cell Program Division of Hematology Ohio State University Columbus, OH United States; 10 Division of Hematology Duke University School of Medicine Durham, NC United States; 11 Division of Pediatric Hematology/Oncology Duke University School of Medicine Durham, NC United States; 12 Department of Internal Medicine East Carolina University Greenville, NC United States; 13 Sickle Cell 101 San Jose, CA United States; 14 Center for Research on Media, Technology, and Health University of Pittsburgh Pittsburgh, PA United States

**Keywords:** sickle cell anemia, sickle cell disease, pain, depression, depressive symptoms, quality of life, digital, mHealth, eHealth, CBT, cognitive behavioral therapy, education, mobile phone

## Abstract

**Background:**

Patients with sickle cell disease (SCD) experience significant medical and psychological stressors that affect their mental health, well-being, and disease outcomes. Digital cognitive behavioral therapy (CBT) has been used in other patient populations and has demonstrated clinical benefits. Although evidence-based, nonpharmacological interventions for pain management are widely used in other populations, these treatments have not been well studied in SCD. Currently, there are no adequately powered large-scale clinical trials to evaluate the effectiveness and dissemination potential of behavioral pain management for adults with SCD. Furthermore, some important details regarding behavioral therapies in SCD remain unclear—in particular, what works best for whom and when.

**Objective:**

Our primary goal is to compare the effectiveness of two smartphone–delivered programs for reducing SCD pain symptoms: digital CBT versus pain and SCD education (Education). Our secondary goal is to assess whether baseline depression symptoms moderate the effect of interventions on pain outcomes. We hypothesize that digital CBT will confer greater benefits on pain outcomes and depressive symptoms at 6 months and a greater reduction in health care use (eg, opioid prescriptions or refills or acute care visits) over 12 months.

**Methods:**

The CaRISMA (Cognitive Behavioral Therapy and Real-time Pain Management Intervention for Sickle Cell via Mobile Applications) study is a multisite comparative effectiveness trial funded by the Patient-Centered Outcomes Research Institute. CaRISMA is conducted at six clinical academic sites, in partnership with four community-based organizations. CaRISMA will evaluate the effectiveness of two 12-week health coach–supported digital health programs with a total of 350 participants in two groups: CBT (n=175) and Education (n=175). Participants will complete a series of questionnaires at baseline and at 3, 6, and 12 months. The primary outcome will be the change in pain interference between the study arms. We will also evaluate changes in pain intensity, depressive symptoms, other patient-reported outcomes, and health care use as secondary outcomes. We have 80% power to detect a difference of 0.37 SDs between study arms on 6-month changes in the outcomes with 15% expected attrition at 6 months. An exploratory analysis will examine whether baseline depression symptoms moderate the effect of the intervention on pain interference.

**Results:**

This study will be conducted from March 2021 through February 2022, with results expected to be available in February 2023.

**Conclusions:**

Patients with SCD experience significant disease burden, psychosocial stress, and impairment of their quality of life. CaRISMA proposes to leverage digital technology and overcome barriers to the routine use of behavioral treatments for pain and depressive symptoms in the treatment of adults with SCD. The study will provide data on the comparative effectiveness of digital CBT and Education approaches and evaluate the potential for implementing evidence-based behavioral interventions to manage SCD pain.

**Trial Registration:**

ClinicalTrials.gov NCT04419168; https://clinicaltrials.gov/ct2/show/NCT04419168.

**International Registered Report Identifier (IRRID):**

PRR1-10.2196/29014

## Introduction

### Background

Sickle cell disease (SCD) is a genetic hemoglobinopathy disorder that predominantly affects those of African descent in the United States [1,2]. Adult patients living with SCD have acute and chronic complications, including daily chronic pain and recurrent, unpredictable, vaso-occlusive episodes of pain, which often require immediate medical attention [3]. These complications have a significant impact on patients’ daily functioning, health-related quality of life (HRQoL), and mental health [1-5]. In addition, acute and chronic pain as well as depression have been associated with increased health care use and/or premature death [6-12].

Current standards for pain management in patients with SCD are inadequate [13,14]. There is a general overreliance on the use of opioids for pain management among providers and patients, despite the known physical and psychological consequences [15-18]. It is estimated that up to 87% of adults and 44% of children aged younger than 6 years with SCD are prescribed opioids [19], despite the lack of data to support their long-term efficacy. The chronic use of daily opioids is associated with side effects and hyperanalgesia, which in turn likely contribute to increased pain [20] and worse HRQoL [21]. There is a pressing need for effective, nonpharmacological interventions to optimize chronic pain management in patients with SCD [22].

Cognitive behavioral therapy (CBT) has been used in other patient populations, resulting in clinical benefits [23-27]. However, even with the broad use of CBT in other pain populations, its use has not been incorporated into pain management plans for individuals with SCD. Multiple barriers have prevented patients with SCD from receiving quality CBT pain services, including a lack of access, limited local availability, expected cost or copayments, and the stigma associated with seeing a mental health provider or specialist.

Advances in technology have had a major impact on the delivery of psychosocial treatments. Although limited access to the internet among minority populations has been problematic in the past, the emergence of mobile technology has helped bridge the *digital divide*. Many patients with SCD have reported wide access to or ownership of personal or mobile devices [28,29]. There has also been growing evidence to support the feasibility, acceptability, and effectiveness of mobile health interventions in patients with chronic medical conditions [30-32], including SCD [33]. For all these reasons, patients with SCD may greatly benefit from digital behavioral interventions that can be accessed on computers or mobile phones [34,35].

Despite the potential benefit of integrating digital CBT into routine SCD care, there are currently no large-scale trials demonstrating the benefits of this intervention approach in this population. In fact, no adequately powered clinical trials have demonstrated the effectiveness and dissemination potential of any behavioral pain management approach for adults with SCD [22,36-38]. There is also limited evidence to guide health care providers on which nonpharmacological pain management strategies are feasible, acceptable, and effective for adult patients with SCD. Therefore, there is a need for an adequately powered pragmatic study to evaluate whether digital behavioral pain treatment strategies are effective and can be implemented at scale into routine SCD care in real-world settings.

### Objectives

The primary objective of the CaRISMA (Cognitive Behavioral Therapy and Real-time Pain Management Intervention for Sickle Cell via Mobile Applications) trial is to compare the effectiveness of two mobile phone–delivered programs for reducing SCD pain symptoms at the 6-month follow-up: digital CBT versus pain and SCD education (Education). We will also evaluate the sustainability of the intervention effects at the 12-month follow-up. The secondary objective is to assess whether baseline depression symptoms moderate the effect of interventions on pain outcomes. We hypothesize that digital CBT will confer greater improvement in pain interference, pain intensity, and depressive symptoms at 6 months compared with Education. We also hypothesize that digital CBT will confer a greater reduction in health care use (eg, opioid prescriptions or refills or acute care visits) over 12 months compared with Education.

## Methods

### Study Design

CaRISMA is a multisite, randomized, pragmatic, comparative effectiveness trial that will be conducted at 6 comprehensive sickle cell centers and 4 community-based organizations (CBOs). A total of 350 adults with SCD who report chronic pain and/or use long-acting or daily opioids will be enrolled and randomized in a 1:1 ratio to receive either a digital CBT program tailored for adults with SCD (CBT) or pain and SCD education (Education) on their mobile phones for 12 weeks. Both programs will use identical mobile-based technology platforms, with the only difference being the content provided. The focus of the digital CBT program is to teach behavioral coping skills through participants’ *seeing and doing*, whereas the pain education arm focuses on improving self-management through participants’ *learning and knowing* more about pain and SCD.

#### Inclusion and Exclusion Criteria

English-speaking adults with any SCD genotype who are 18 years of age or older reporting chronic pain (ie, pain at least 4 days a week over the past 3 months or longer) and/or being prescribed long-acting or daily opioid medication for pain will be eligible to participate. As the intervention content and presentation were specifically designed for adults, age was restricted to individuals aged 18 years or older. Patients who do not meet the chronic pain criteria or simply do not want to participate in an intervention arm of the study have the option to complete the battery of questionnaires at baseline and each time they return to the clinic for a routine follow-up visit. This convenience sample of nonintervention patients will be used for exploratory comparisons and is not included in the target sample size of 350.

Individuals with cognitive dysfunction or low literacy may not benefit from all components of the intervention. During the electronic consent process, potential participants will be required to answer six consent comprehension questions correctly. These questions aim to ensure that all enrolled participants understand the study protocol and have the appropriate level of literacy and cognitive functioning to benefit from the intervention. Any participant who fails the consent comprehension assessment will be excluded from participation at the time of screening but may be rescreened in 3 months.

A smartphone is required for participation. Otherwise, eligible patients who do not own a smartphone will be provided one with cellular and data service for 12 months as part of the study.

#### Recruitment, Enrollment, and Randomization

The target sample size for this study is 350 participants. We will implement a hybrid strategy of in-person and web-based enrollment to ensure that we recruit a representative spectrum of the adult SCD population, regardless of their location. In-person recruitment will be from six clinical academic sites: University of Pittsburgh Medical Center, Duke University, Johns Hopkins University, Ohio State University, University of Illinois at Chicago, and East Carolina University. Remote, web-based recruitment will be done through the following CBO partners: Sickle Cell 101 (SC101), Sickle Cell Community Consortium (SCCC), Sickle Cell Warriors (SCWarriors), and Children’s Sickle Cell Foundation.

#### In-Person Recruitment at Clinical Sites

At each of the enrollment sites, a tablet-administered screening tool and best practice alerts will be used to identify patients with SCD who have chronic pain, as indicated by self-reports. The tool will also flag patients with a chronic pain diagnosis or those who have been prescribed chronic opioid therapy for pain. Each clinical site will screen approximately 90-100 patients, and we expect to approach and screen 540-600 potential participants across all six sites.

#### Web-Based Recruitment Through Community Partners

Our partnering CBOs have created an active community that includes more than 10,000 patients with SCD. For this study, SC101, SCWarriors, Children’s Sickle Cell Foundation, and SCCC and their partnering organizations will seek potential participants via message blasts on their CBO websites, social media groups, and email listservs and at patient or family meetings. Interested patients will access a short web-based screener on a mobile device to confirm their eligibility. Potential participants must be able to read the study consent and correctly answer comprehension questions to ensure their understanding of the study goals and procedures. Our group has published data on the use of this electronic consent process in a clinical study [39] and a large multisite trial of digital CBT [40], which is also used in other ongoing studies.

#### Participant Incentives

Participants who enroll in the intervention, but do not receive a study smartphone, will instead receive payment to cover the cost of smartphone data or use while on the study, for a total of US $250. This will be paid in increments throughout the participants’ 12-month participation in the study. At baseline, participants will receive US $75 after completing all baseline questionnaires, accessing and beginning the intervention program, and making one phone contact with their health coach. At each follow-up (ie, 3-month, 6-month, and 12-month time points), participants will receive US $50 after completing the questionnaires for the follow-up assessment. An additional US $25 will be given to those who complete all four follow-ups throughout the 12-month study period. Participants who are given a smartphone will not receive any monetary compensation. Participants in the nonintervention comparison group will not receive payment.

#### Randomization

Upon completion of eligibility, participants will be randomly assigned to either digital CBT or Education. Permuted block randomization will be stratified by study center (eg, one of the six clinical sites or from a community partner) in an effort to control for site-specific disease education and treatment approaches. The randomization schema was created by the lead statistician in the data coordinating center and integrated into the web-based data capture system.

### Study Procedures

**Figure 1:**
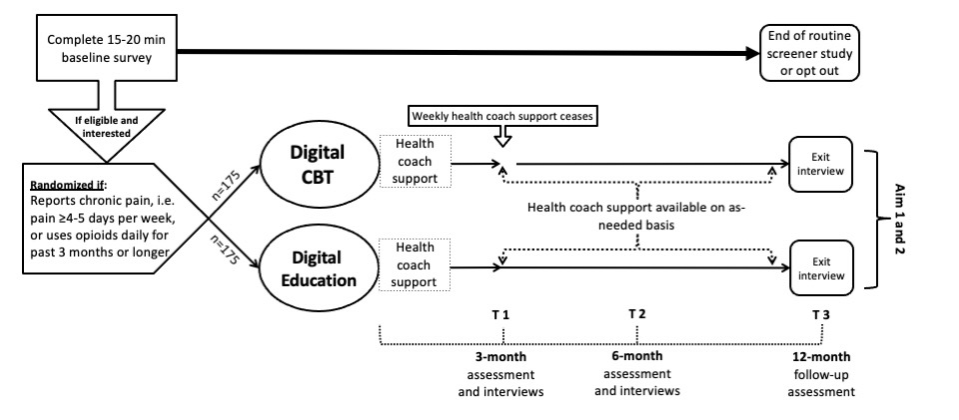
CaRISMA (Cognitive Behavioral Therapy and Real-time Pain Management Intervention for Sickle Cell via Mobile Applications) study design with two intervention arms (digital cognitive behavioral therapy vs education) and 12 months of follow-up for all participants. CBT: cognitive behavioral therapy.

In the first 3 months of the study, intervention participants will be asked to complete the modules for their assigned study arm, that is, digital CBT or Education (Figure 1). The recommended progression is one module per week over 12 weeks, which will give participants time to practice the techniques they learn in each module.

At 3-, 6-, and 12-month follow-up, participants will receive a link to a web-based survey tool to complete the study assessments, including Painimation, the Sickle Cell Self-Efficacy Scale (SCSES), Patient Reported Outcomes Measurement Information System measures, Adult Sickle Cell Quality of Life Measurement Information System measures, Patient Health Questionnaire-9 (PHQ-9), and General Anxiety Disorder Scale-7 (GAD-7). Between these follow-up assessments, participants will be expected to continue reporting their daily pain intensity diaries through a web-based survey tool that they can access on their smartphone app. A summary of the key study measures and outcomes is presented in Table 1.

**Table 1:** Schedule of study outcomes.

Outcome and measure			Month of study						
			0		3		6		12
**Primary**									
	Patient Reported Outcomes Measurement Information System Pain Interference	✓		✓		✓		✓	
**Secondary**									
	Daily pain intensity (mobile web app) or Painimation^a^	✓		✓		✓		✓	
	ASCQ-ME^b^ Emotional Functioning and Social Impact scales	✓		✓		✓		✓	
	Depressive symptoms (Patient Health Questionnaire-9)	✓		✓		✓		✓	
	Generalized Anxiety Disorder Scale-7	✓		✓		✓		✓	
**Medical**									
	Health care use (PCORnet): opioid prescriptions, emergency department visits, and hospitalizations							✓	
**Process evaluation**									
	Sickle Cell Self-Efficacy Scale	✓		✓		✓		✓	
	Program engagement and treatment dose	✓		✓		✓		✓	

^a^Measured daily for 12 months.

^b^ASCQ-Me: Adult Sickle Cell Quality of Life Measurement Information System.

For intervention participants, in addition to the quantitative assessments, the University of Pittsburgh Qualitative, Evaluation, and Stakeholder Engagement (Qual EASE) Research Services will conduct a set of qualitative interviews at the end of the study. The goal of these interviews is to further understand the lived experience of patients in the trial and determine which intervention works best for whom and when. Qual EASE Research Services will select and interview 24 patients in each treatment group, stratified by high or low depression scores, for a total of 48 interviews (ie, 12 digital CBT patients with a PHQ-9 score ≤10, 12 digital CBT patients with a PHQ-9 score >10, 12 digital Education patients with a PHQ-9 score ≤10, and 12 digital Education patients with a PHQ-9 score >10). This distribution will allow us to qualitatively compare the experiences of patients with different depression levels and explore quantitative findings related to intervention efficacy in patients with various levels of depression severity. The patient sample size has been selected to allow for a high likelihood of reaching thematic saturation regarding patient experience [41].

### Description of Study Arms

**Figure 2:**
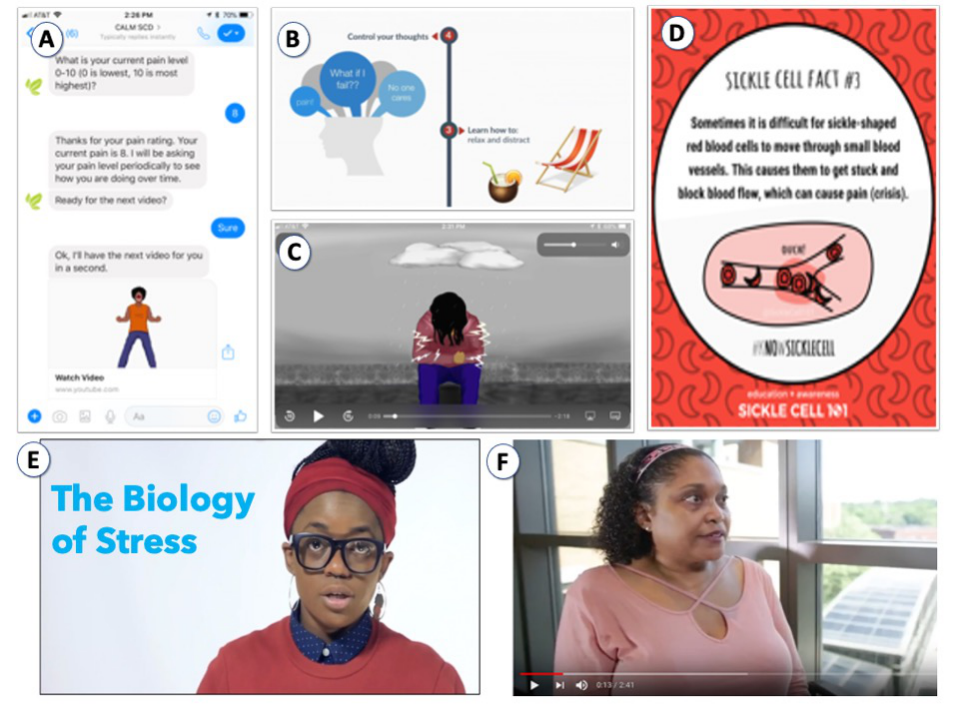
Screenshots of (A) the interactive chatbot that asks users questions and pushes appropriate content. (B) and (C) show examples of cognitive behavioral therapy content that teaches control of the negative thoughts and mood contributing to pain. (D) Example of educational content, curated by Sickle Cell 101, which teaches general facts about sickle cell disease and pain. (E) and (F) show videos of adults with sickle cell disease teaching content and talking about their experiences with stress.

There are three main components of the CaRISMA program (Figure 2): the chatbot app, a health coach, and an online support group. Both study arms will receive chatbot app interventions. Using a scripted chatbot and conversational interface, the chat component of the program provides 24/7 chat-style interactions to assess participants’ needs and deliver personalized content to them. As users move through the chatbot, they can view their progress and gain access to all of the tools and lessons they have unlocked. They also have the ability to view their status and the badges they have received for specific accomplishments. The intervention encourages users to visit a publicly accessible social media group and virtual meet-up activities promoted by the CaRISMA program.

#### Digital CBT Arm

The digital CBT program for pain management will teach users how to recognize negative thoughts and emotions, use cognitive and problem-solving skills, and apply coping behaviors, such as distraction, activity scheduling, and relaxation. All of the video content on the CBT arm is co-designed with, delivered by, and features adults living with SCD. The digital CBT arm emphasizes skills acquisition and learning through practice; thus, the program involves homework assignments and challenges as well as continued check-ins with a health coach who helps reinforce CBT skills and encourages practice and program engagement. The digital CBT program also gives users access to a study-associated Facebook page where they can discuss with other patients the issues that they faced and what skills were or could be used to address them. This intervention is consistent with the tailored behavioral services that patients would receive individually or as a group when working with a psychologist or behavioral pain specialist.

#### Digital Education Arm

The digital Education intervention, also delivered via the CaRISMA chatbot, is focused on pain and SCD education. The Education program teaches users about chronic pain, healthy lifestyle tips (eg, nutrition and exercise), and facts about SCD. The emphasis is on knowledge acquisition and gives users an opportunity to apply what they have learned through brief quizzes and discussions with the health coach and their social network. All of the education program content is developed and delivered by SC101 and features their community members, adults with SCD. This program is consistent with the education that patients and families would receive with a patient educator or what is currently provided via the web and social media through two of our community partners, SCWarriors and SC101. Users in the Education arm will also be asked to access publicly accessible social media groups and virtual meet-up activities.

### Health Coaches

Both study arms will have access to a health coach. Health coaches all have minimum a college education with some background in community health, patient advocacy, or clinical psychology. Some health coaches are adults who live with SCD. The number of health coaches is expected to vary throughout the 3-year study, with 3-5 coaches active at any given time, some of whom will be adults with SCD. All health coaches undergo approximately 8 hours of training in CBT, motivational interviewing, acceptance and commitment therapy, problem-solving techniques, general counseling strategies, and advanced education on SCD. In addition to the initial training, over the course of the study, health coaches will attend a weekly group supervision that includes role-playing and review of interactions (phone or text message) with participants and weekly one-on-one supervision sessions, with a clinical psychologist.

The primary function of the health coach is to provide emotional and informational support on a weekly basis. The health coach will reinforce the use of skills and interventions learned within the program. The health coach will touch base with participants once a week, ideally over the phone, but texting will also be acceptable. In addition, health coaches may send personalized texts in between weekly meetings to help participants stay motivated. After 12 weeks, participants will no longer be required to engage with their health coach; however, if they would like to remain in contact with their health coach, they will be able to reach out and make an appointment.

To maintain intervention continuity and fidelity, health coaches will participate in weekly supervision sessions led by a masters-level psychologist and the study principal investigator (PI) for review and discussion of participant interactions. Telephone calls will be periodically recorded, at random, for audits and reviews during supervision sessions. All text message communications will be sent from a common account, and the content of these messages is monitored and periodically audited.

### Study Population: Recruitment and Retention

We will overrecruit by 15% to account for potential dropouts and loss to follow-up. However, every effort will be made to maintain participation in the study groups and obtain all posttreatment measures from all participants. Participants will receive a gift card for each outcome assessment completed. We will ask for multiple phone numbers, and we will use emails, text messages, and phone calls to remind participants of the various web-based assessments. Strategies to promote adherence include an appealing and engaging user-centered program, gamification elements (eg, positive feedback loops and short-term goals), and social elements (eg, peer support). The characteristics of adherent versus nonadherent participants will be assessed for systematic differences, which if found will be examined with sensitivity analyses to determine their effect on outcomes.

### Study Outcomes and Measures

#### Primary Pain Outcome: Pain Interference

Patient Reported Outcomes Measurement Information System Pain Interference [42] assesses the effect of patient-reported pain on relevant aspects of a person’s life and may include the extent to which pain hinders engagement with social, cognitive, emotional, physical, and recreational activities. This measure will only be completed at baseline and 3, 6, and 12 months.

#### Secondary Pain Outcome: Daily Pain Intensity

Participants will be asked to enter their daily pain via a mobile website. They will receive reminder notifications via text messages for 2-week periods at baseline and 3, 6, and 12 months. However, between these automated-reminder periods, participants will be encouraged to continue entering their pain scores on the mobile pain web app.

#### Other Secondary Outcomes

##### Painimation

**Figure 3:**
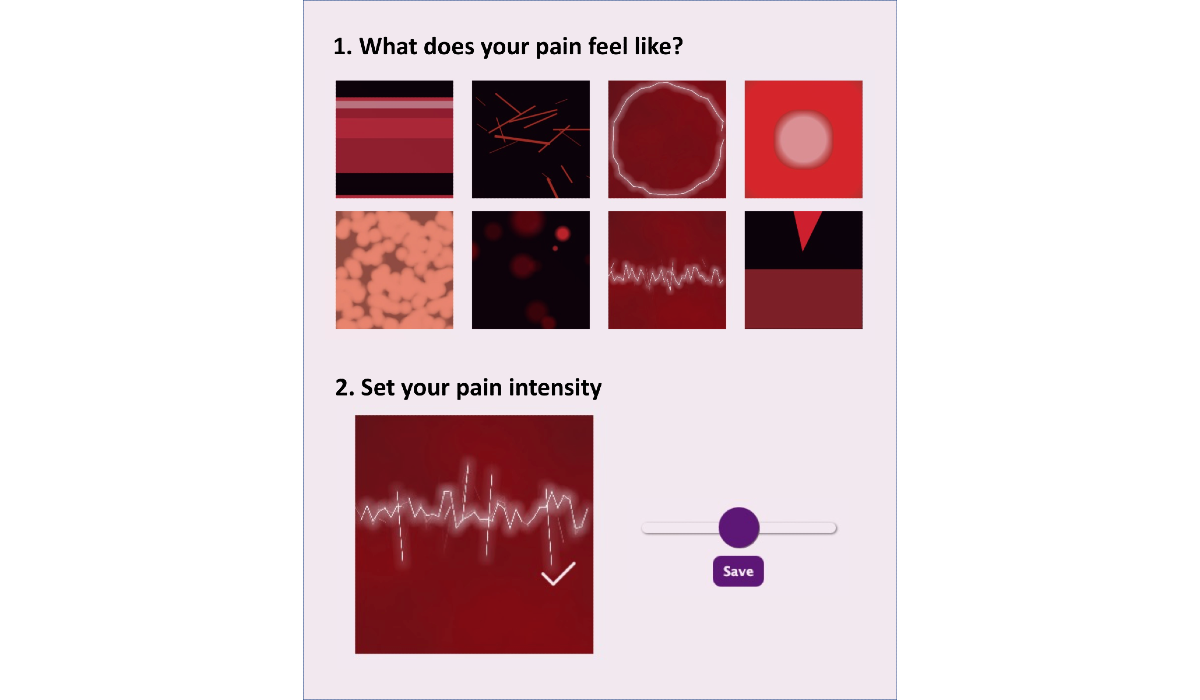
The Painimation screen allows users to select and adjust up to three animations to reflect pain quality and intensity.

Painimation is an electronic pain assessment tool that allows users to better communicate pain symptoms [39]. Patients are provided with a selection of animations (*painimations*) that they use to describe the quality of their pain. The painimations can be adjusted to reflect pain intensity. Screenshots of the Painimation app illustrate the splash screen, paintable body image, and selection of painimations to indicate the quality and intensity of pain (Figure 3).

##### Medical Outcomes

For patients recruited at one of the six clinical sites, we will evaluate objectively measured opioid medication prescriptions and refills and emergency department visits or hospitalizations for pain episodes. We will work in collaboration with PCORnet to collect data retrospectively (12 months before enrollment) and prospectively (12 months after enrollment) from patients’ electronic health records. These data will allow us to track opioid medication use, health care use, and laboratory values (eg, hemoglobin level), as well as other key clinical outcomes. For patients recruited from our CBO partners or on the web, we will only evaluate their medical records to confirm their SCD diagnosis if they do not receive their SCD care at one of the participating clinical sites. 

##### Current Opioid Misuse Measure

Current Opioid Misuse Measure [43] is a self-report measure to monitor indicators of current aberrant drug-related behaviors in patients with chronic pain on opioid therapy. This measure will be completed only at baseline and 12 months.

##### Patient Health Questionnaire

PHQ-9 assesses the degree of depression severity [44]. The PHQ-9 total score is for nine items, all rated as *0* (not at all) to *3* (nearly every day), with total scores ranging from 0 to 27. Scores of 5, 10, 15, and 20 represent cut-off points for mild, moderate, moderately severe, and severe depression, respectively [45]. This measure will only be completed at baseline and 3, 6, and 12 months.

##### Generalized Anxiety Disorder Scale-7

GAD-7 evaluates the severity of anxiety [46]. The GAD-7 total score for the 7 items ranges from 0 to 21. Scores of 5, 10, and 15 represent the cut-off points for mild, moderate, and severe anxiety, respectively [47]. This measure will only be completed at baseline and 3, 6, and 12 months.

##### Adult Sickle Cell Quality of Life Measurement Information System Emotional Functioning and Social Impact Scales

The ASCQ-ME emotional functioning and social impact quality-of-life measure was specifically designed for SCD and evaluates the health care experience of patients with SCD, emotional response to stress, and social relationships [48]. These measures will only be completed at baseline and 3, 6, and 12 months.

##### Sickle Cell Self-Efficacy Scale

SCSES is a self-report measure to assess the ability of patients with SCD to function on a day-to-day basis and manage their SCD symptoms [49]. This measure will only be completed at baseline and 3, 6, and 12 months.

### Adverse Events

**Figure 4:**
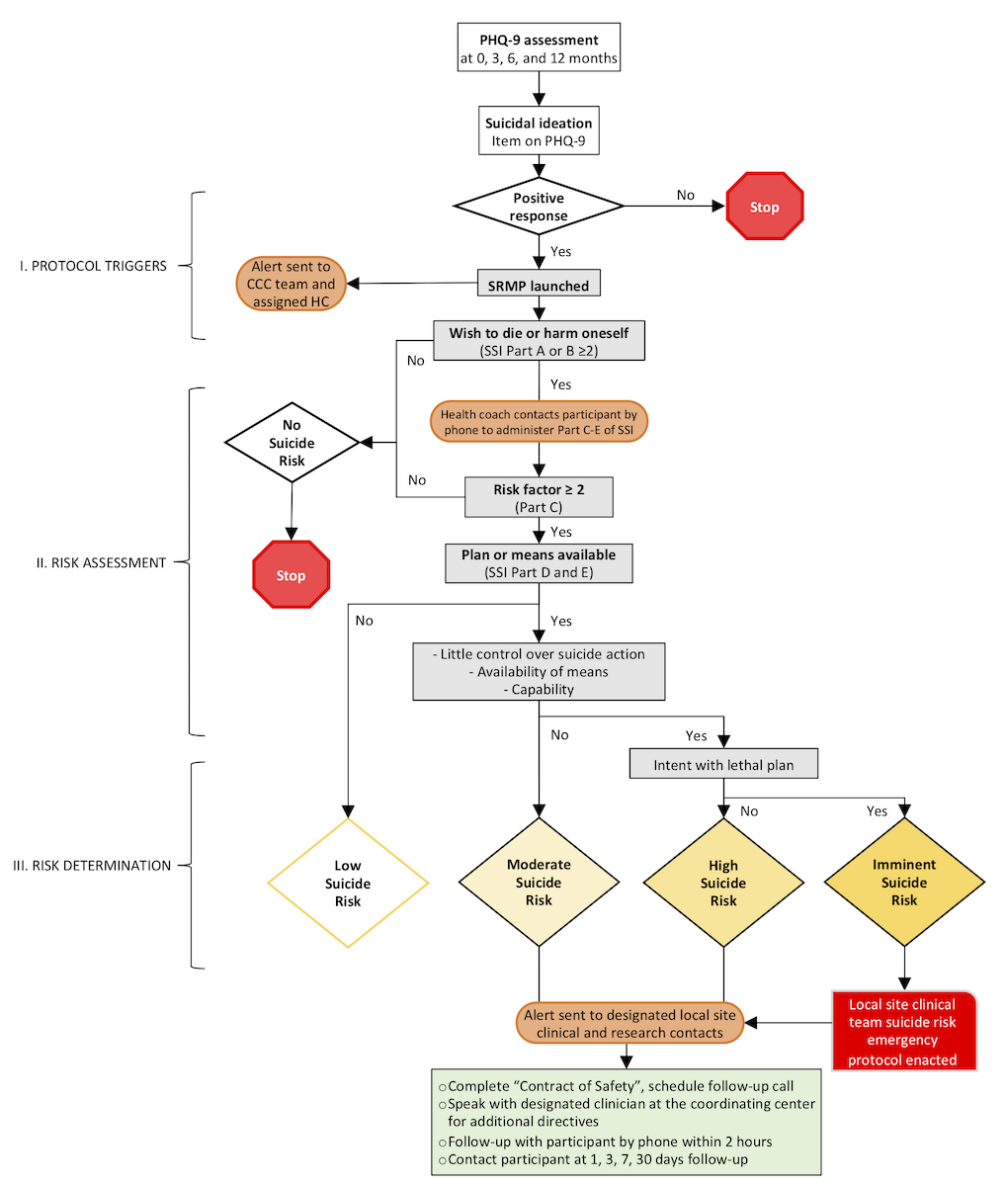
Flow diagram for the Suicide Risk Management Protocol. CCC: Clinical coordinating center; HC: Health coach; PHQ-9: Patient Health Questionnaire-9; SRMP: Suicide Risk Management Protocol; SSI: Suicide Severity Index.

We adapted the following definition to address the negative effects of internet interventions [50] and face-to-face behavioral treatment [51]: adverse events consist of negative events that may emerge from treatment and are perceived as adverse by the patient, causing the deterioration of target symptoms and/or negative experiences that extend beyond the completion of treatment. Examples include increased anxiety during CBT training or being embarrassed by revealing negative thoughts and insecurities to the health coach. Adverse events could also reveal negative effects directly attributable to treatment, providing our team and other researchers with information on possible mechanisms underlying these negative effects. The health coaches will routinely assess for increasing negative affect, and the GAD-7 will be administered at 3, 6, and 12 months to assess for increasing anxiety. In addition, we developed a suicide risk management protocol to illustrate risk triggers, assessments, and determinations (Figure 4).

### Sample Size Determination

For the primary test of comparative effectiveness of digital CBT versus mobile education (m-Education) for the trial (aim 1), the power calculation is based on the comparison of intervention groups for the main outcome of the 6-month change in pain interference. Specifically, our sample size of 350 participants enables 80% power to detect a difference of 0.37 SDs between study arms on 6-month changes. This detectable effect is also applicable to key secondary outcomes, such as pain intensity and depression, as measured by the PHQ-9. Our calculations account for 15% attrition at 6 months.

### Statistical Analysis

All primary and secondary analyses will be preceded by descriptive analyses of the baseline and clinical characteristics. Summary statistics will include means and SDs for continuous variables and sample proportions for categorical variables. Median and IQR values will accompany nonnormal, continuous variables. The results will be presented both within and across study arms. All analyses will follow the intention-to-treat approach.

#### Analysis of Primary and Secondary Pain Outcomes

Linear mixed models will be employed for the primary outcome of pain interference as a function of time, study arm (digital CBT vs Education), time×study arm interaction, study site, and baseline depression level (PHQ-9≤10 vs PHQ-9>10). We will account for multiple observations for each participant by including a random effect for the subject. In addition, baseline variables with large, clinically meaningful between-arm differences will be included as covariates in the secondary analyses. Contrasts will be estimated to assess the impact of digital CBT and education intervention on 6-month improvements in pain intensity. As a secondary investigation, we will use the same linear mixed models to address whether the 6-month improvements are sustainable for 12 months. The analytic strategy for the key secondary outcome of pain intensity will be identical to that of the primary outcome.

As the confirmation of a sickle cell diagnosis may not be made immediately after randomization, we will conduct an a priori sensitivity analysis restricting eligible participants with the confirmation of SCD.

#### Analysis of Secondary Outcomes

Secondary outcomes such as PHQ-9, Current Opioid Misuse Measure, GAD-7, Adult Sickle Cell Quality of Life Measurement Information System, and SCSES will be analyzed using similar linear mixed models with time, study arm, time×study arm interaction, study site, and baseline depression level as fixed covariates and a random effect for each subject. Health care use will be compared between study arms using generalized linear models to account for count data (ie, Poisson regression) for each of the following: the number of opioid prescriptions, number of emergency department visits, and number of hospitalizations over 12 months after study entry. Predictors will include the study arm, study site, and baseline depression level. The corresponding health care use in the 12 months before study entry will be entered as a covariate in each model. We will offset each model by the participant’s time in the study.

#### Subgroup Analysis

We will examine the heterogeneity of the treatment effect to determine whether the intervention works better for some than for others. Our prespecified analysis plan will examine differences in pain interference between patients with high (PHQ-9>10) and low (PHQ-9≤10) depression. We will augment the primary analysis models for pain interference with relevant main effects for the baseline depression level and 2- and 3-way interactions with the study arm and time. Of primary interest is the significance of the 3-way interaction (time×study arm×baseline depression level). If significant, we will present the treatment effect estimates and 95% CIs within each subgroup.

#### Mediation Analysis

For coping skills, self-efficacy, and program engagement or dose measures, changes in scores from baseline to 3 months and from baseline to 6 months will be tested as potential mediating variables. We will use the framework of Kraemer et al [52] to calculate effect sizes, accounting for the impact of the potential mediator variable. In addition to modeling each mediator as a function of time, study arm, time×study arm interaction, study site, and baseline depression level (PHQ-9 ≤10 vs PHQ-9 >10), we will augment the original primary analytic models by including the potential mediator as a covariate.

#### Addressing Missing Data

We will minimize missing data by using an electronic data collection system and by contacting participants when data are not entered in a timely fashion. We will attempt to characterize the missingness mechanism (missing completely at random, missing at random, or not missing at random) by comparing rates of missingness or attrition between study arms. In addition, we will compare baseline characteristics between participants with and without missing outcome data. If we conclude that our missingness is random, then our likelihood-based approach for the primary analyses will address this. Otherwise, if the missingness can be characterized as nonignorable (not missing at random), we will use approaches such as joint modeling or shared parameter models to produce unbiased estimates of treatment effects. All reasons for dropout or other missing values will be entered by the research staff or community groups (if encountering the patient during community activities) and will be tabulated and summarized, and results will be reported using the CONSORT (Consolidated Standards of Reporting Trials) diagram. Patients who stop reporting data in the app will receive inquiry text messages or phone calls.

#### Qualitative Analysis

Interviews will be conducted and analyzed by Qual EASE Research Services in the data center of the Center for Research on Health Care at the University of Pittsburgh. An analysis of the interviews will combine thematic analysis and the constant comparison method [53]. Codes will be developed via open coding of the transcripts to determine topics and themes that emerged in the interview transcripts, but input on topics or themes that the study team anticipates being relevant will also be solicited, resulting in simultaneous inductive and deductive development of the codebook. Once the codebook is finalized, 2 data analysts from Qual EASE will be trained using the codebook, following which both coders will independently code 25% of the transcripts. Coding will then be compared to calculate Cohen κ intercoder reliability scores. Any coding discrepancies identified during this comparison will be adjudicated until full agreement is achieved. After satisfactory intercoder reliability (Cohen κ>0.6) is achieved, the primary coder will code the remaining transcripts. The completed coding will form the basis of a thematic analysis of the data and a constant comparative analysis to compare the experience of patients with depression levels.

### Supporting Documentation and Operational Considerations

#### Regulatory, Ethical, and Study Oversight Considerations

The procedures set out in this protocol pertaining to the conduct, evaluation, and documentation of this study are designed to ensure that the investigator abides by Good Clinical Practice guidelines and the guiding principles detailed in the Declaration of Helsinki. The CaRISMA trial will rely primarily on the review of the Institutional Review Board, University of Pittsburgh Human Research Protection Office, while establishing an agreement that all site-specific institutional review boards (IRBs) will review their informed consent documents to ensure local concerns are adequately addressed. Modifications to the study protocol will not be implemented by the investigator without discussion and agreement by the data coordinating center and the study sponsor (ie, Patient-Centered Outcomes Research Institute [PCORI]). However, the investigator may implement a deviation from, or a change in, the protocol to eliminate an immediate hazard(s) to the trial patients without prior independent ethics committee or IRB approval or favorable opinion. Any deviations from the protocol must be explained and documented by the investigator. The PI at each institution or site will be responsible for ensuring that all the required data will be collected and properly documented.

Monthly meetings with the study co-PIs, clinical site PIs, community partners, and coinvestigators will help uncover and address study-related issues. These issues will be brought to quarterly data safety monitoring board meetings, which will be responsible for overseeing the trial’s progress; making recommendations regarding the safety and benefit of continuing or stopping the trial; ensuring the trial is operating in accordance with similar trials, information from which will be gathered and reviewed; disseminating status and progress updates from the trial to sponsors, funders, and other groups; and making recommendations about how to present the results of the trial on a wider scale to a broader audience.

#### Informed Consent Process

Before performing any of the research study procedures or interventions, the participants must provide informed consent. Informed consent is a process that is initiated before the individual agrees to participate in the study and continues throughout the individual’s study participation. The consent process will occur over the internet and is self-guided. Potential participants can access the consent website via their own electronic device or in a clinical setting via a tablet computer station (kiosk). A video presentation will explain the study to the potential participants in a language understandable to participants, providing all pertinent information (purpose, procedures, risks, benefits, alternatives to participation, etc). A comprehension test will be conducted to ensure participants’ understanding of the study goals and procedures. In addition, the most pertinent consent language will be presented on separate webpages where the user must click a button indicating acknowledgment and understanding of the content to advance to the next screen. Each segment of the consent is presented in concise, easy-to-understand language. Finally, the full consent document will be presented in the form of a PDF on the screen for the potential participant to read in its entirety.

#### Study Discontinuation and Closure

If this study is prematurely terminated or temporarily suspended, the PI will promptly inform ongoing study participants, the IRB, and the sponsor or funding agency. The PI will also provide the reason(s) for the termination or temporary suspension and describe the process of handling consented or enrolled participants in the event that the study is prematurely terminated.

#### Confidentiality and Privacy

All participant information, including contact information, questionnaires, and clinical information, will be monitored by the study staff and be available only to them in a Health Insurance Portability and Accountability Act–compliant database. Case report forms and other electronic data will be stored in password-protected files. Only authorized study staff will have access to the study data. Study reports will be deidentified and present findings in the aggregate (or by treatment group).

## Results

All study investigators from academic clinic sites and all partners from CBOs convened in May and November 2020 to discuss study progress and finalize plans for study initiation. The IRB at the University of Pittsburgh Human Research Protection Office approved the protocol for the CaRISMA study in May 2020. Owing to COVID-19, enrollment was postponed for approximately 6 months. Study enrollment officially started in March 2021, and the study will be conducted through October 2022, with results expected to be available in February 2023. The study was registered at ClinicalTrials.gov (NCT04419168).

## Discussion

### Principal Findings

Patients living with SCD have several complications, including daily chronic pain and recurrent, unpredictable vaso-occlusive episodes that often require immediate medical attention [3]. These complications lead to significant impairment in patients’ HRQoL across their lifespan, particularly mental and psychosocial well-being. Current standards for pain management in SCD are unsatisfactory and primarily focus on opioids, with little evidence for nonpharmacological interventions in this population [27,28]. The increasing use of opioids has led to several physical and psychological consequences, and the development of effective, nonpharmacological interventions is essential to optimize pain management in adults with SCD [22].

Behavioral interventions have been shown to be efficacious [54-56], but the lack of widespread availability of therapists and CBT-trained clinicians has made implementation into routine care challenging, especially in minority populations, such as patients with SCD. Despite strong evidence supporting the efficacy of digital CBT [23-27], its use remains limited in SCD. With the growing access to mobile and personal technology [28,29], digital CBT has great potential to address patients’ need for evidence-based, user-centered, and rigorous behavioral programs [34,35].

CaRISMA is a large-scale, multi-institution, adequately powered pragmatic study in partnership with CBOs aimed at comparing the effectiveness of two evidence-based behavioral approaches to pain management: CBT and Education. This study will ultimately determine how digital behavioral pain treatment strategies can be effectively implemented at scale in routine care for adults with SCD in real-world settings. CaRISMA has the potential to inform behavioral nonpharmacological pain management approaches with strategies that are feasible, acceptable, and effective, which medical providers can offer to their adult patients with SCD [22,36-38].

### Conclusions

The CaRISMA trial will fill a knowledge gap in the SCD literature by evaluating the effectiveness of two remotely delivered pain management programs for adults with SCD: digital CBT and digital Education. In this trial, we will also evaluate the sustainability of intervention effects over time and other related key aspects of pain management, such as depression and emotional and psychosocial well-being. Addressing these questions will be essential to inform future research directions of digital behavioral interventions, not only for adults with SCD but also for children and adolescents with SCD as well as other chronic pain populations. If digital CBT demonstrates effectiveness in this trial, future dissemination and implementation efforts will be critical to ensure that this intervention is widely available and used by many adult patients with SCD in the United States and worldwide.

## Appendix

Multimedia Appendix 1 Comments from the grant proposal peer-review process with the Patient-Centered Outcomes Research Institute.

Multimedia Appendix 2 Responses to comments from the peer-review process using Patient-Centered Outcomes Research Institute.

## Abbreviations

CaRISMA: Cognitive Behavioral Therapy and Real-time Pain Management Intervention for Sickle Cell via Mobile Applications
CBO: community-based organization
CBT: cognitive behavioral therapy
GAD-7: General Anxiety Disorder Scale-7
HRQoL: health-related quality of life
IRB: institutional review board
PCORI: Patient-Centered Outcomes Research Institute
PHQ-9: Patient Health Questionnaire-9
PI: principal investigator
SC101: Sickle Cell 101
SCCC: Sickle Cell Community Consortium
SCD: sickle cell disease
SCSES: Sickle Cell Self-Efficacy Scale
SCWarriors: Sickle Cell Warriors
Qual EASE: Qualitative, Evaluation, and Stakeholder Engagement
