# Supplementary material for: Comparing the Effectiveness of Education Versus Digital Cognitive Behavioral Therapy for Adults With Sickle Cell Disease: Protocol for the Cognitive Behavioral Therapy and Real-time Pain Management Intervention for Sickle Cell via Mobile Applications (CaRISMA) Study
Source: JMIR Res Protoc. 2021 May 14;10(5):e29014. doi: 10.2196/29014 (PMC8164118; doi:10.2196/29014)
Supplement: Multimedia Appendix 1 [file resprot_v10i5e29014_app1.docx]

Request ID#: PIR-0842

Project Title: iCanCope with Sickle Cell Disease: A Cognitive Behavioral Pain Management Program versus Pain Educat

**PRIVILEGED COMMUNICATION**

Dear Charles Jonassaint:

Thank you for your interest in PCORI and for the submission of your application to the Cycle 2 2018 Assessment of Prevention Diagnosis and Treatment Options program.

Your application was discussed during the in-person merit review panel. We are now conducting our preliminary programmatic and administrative review to assist us in evaluating your application. This email should not be construed as approval of the application budget or project plan; instead it helps inform our decision on the final set of applications to recommend for funding.

During our review we have identified questions that require additional information related to the project plan and budget for your study.

The questions are listed in detail below. Please address the following issues using  [PCORI Online](https://pcori.force.com/engagement) by **1/29/2019**.

**PROGRAMMATIC**

1. Treatment efficacy: PCORI is primarily interested in comparative clinical effectiveness research (CER), in which two or more interventions with robust prior evidence of efficacy are compared.  It appears that the proposed trial may be closer to an efficacy study.  Please address the existing evidence of efficacy for the specific version of cCBT being used in this study.  If insufficient data are available, please describe the specific differences between cCBT interventions with stronger evidence of efficacy and the program being proposed in the current study, and address why the current intervention represents an improvement over prior cCBT interventions (e.g., Web-MAP).  Similarly, please provide strong primary references to support the efficacy of pain education, and if available, references to support the specific version of pain education being used in this study.  If there are none available, please provide documentation to support the statement that pain education, in general, is "widely available" for patients with SCD (p. 1 of Research Plan).
2. Rationale for selection of intervention and comparator: There is concern regarding whether there is clinical equipoise between the two arms, as pain education/psychoeducation has been used as a control condition in prior CBT efficacy trials.  Please describe the process of selecting pain education as the most appropriate comparator to CBT, and why it was selected over other, more active comparators.  Please include other comparators considered in a tabular format. Also describe the significant "decisional dilemma" addressed by the current comparison for patients, providers, and other stakeholders (i.e., are patients and providers faced with a difficult choice between these two interventions?).
3. Current clinical use of interventions: Please clarify whether either intervention is currently in clinical use for chronic pain or SCD; if not, please describe the extent of prior use, even if pilot work, particularly for the pain education intervention.
4. Industry partners: The role of industry partners Wondros and Robots & Pencils is unclear; specifically, the rationale for and extent of any planned adaptation requires clarification and a stronger rationale.  Please describe in greater detail why any adaptation is warranted, the types of adaptations planned, and how each industry partner will contribute to the study's implementation, with specific attention to whether any modifications made to the existing interventions are expected to increase usability, accessibility, or efficacy.  Also, please address whether Wondros has any prior experience working with the proposed patient population.
5. Primary outcome: Per the funding announcement, PCORI encourages investigators to include well-validated outcome measures, with known psychometric properties.  The pain intensity outcome appears still to be in development.  Further, pain intensity is less frequently used as a primary outcome than functional impairment/disability in many studies of chronic pain populations.  Please consider whether the PROMIS pain measure could be moved from a secondary outcome to a primary or co-primary outcome.
6. Recruitment plan: Please provide additional details to support the feasibility of the recruitment plan, as there is concern that the enrollment and retention rates (estimated attrition rate of 15%) in particular may be overly optimistic, particularly given the proposed population of underserved, minority patients with SCD. This may include providing details such as recruitment rate from prior, similar trials completed by the study team, or in progress at study sites. Please use these data to support the anticipated recruitment rate for the proposed study, including the anticipated number screened, eligible, enrolled, and retained for each study site.
7. Opioid use: The application lacks details about the role of opioids in managing SCD pain. Given the common use of opioid medications in patients with SCD, discuss whether additional opioid-related outcomes of interest could be examined, such as average morphine equivalent dose (MED).

**ADMINISTRATIVE**

- No Administrative Requests -

Please download the “Previous, Current and Pending Support” template available [here](http://www.pcori.org/sites/default/files/PCORI-Previous-Current-Pending-Support-Template.doc): This should be completed for all key personnel and returned as one MS Word attachment in your response to PCORI.

***Please disregard this request if you have already submitted this information to the PCORI staff.*

We recognize that a new budget may be required in order to respond appropriately to the programmatic concerns. If there is a significant impact on the budget then you will need to provide a revised budget taking into account the suggested changes.

To access and respond to the PCORI Information Request please follow the link provided below and log in to PCORI Online.

Access Your PCORI Information Request from the Following URL:

<https://pcori.force.com/engagement/a3J39000000oRln>

**Guidelines for Submission:**

1. Submit document(s) as attachments through PCORI Online only using the link provided.
2. Submit programmatic and administrative responses in separate documents.
3. Submit all response attachments at once using the **Submit Response** button in PCORI Online; do NOT reply to this email with your response and/or attachments.
4. BUDGET SUBMISSION: If an updated budget has been requested, please follow the steps below to submit the updated budget to PCORI.
   - Navigate to Budget List section within PCORI Online. Click on the link to 'Edit' the budget version with Budget Status 'Working Budget – Pending Submission'
   - Edit the budget as necessary
   - Navigate to the 'Budget Summary' page and submit using the 'Submit for Review' button
5. For all other documentation pertaining to this request, please submit in the form of MS Word and Excel only. Do NOT submit PDF documents.

**Responding to the aforementioned requests does not guarantee that an application will be recommended for funding**. All awards recommended for funding will undergo a thorough business review before contract execution.

This email should be treated as confidential and not shared publicly or with others within the organization who are not involved with the preparation of response materials.

If you would like to discuss this letter, please email pfa@pcori.org with available times for a call, and we will coordinate a meeting.

Sincerely,

Contract Management
Program Support and Information Management
Patient-Centered Outcomes Research Institute
