# Supplementary material for: Comparing the Effectiveness of Education Versus Digital Cognitive Behavioral Therapy for Adults With Sickle Cell Disease: Protocol for the Cognitive Behavioral Therapy and Real-time Pain Management Intervention for Sickle Cell via Mobile Applications (CaRISMA) Study
Source: JMIR Res Protoc. 2021 May 14;10(5):e29014. doi: 10.2196/29014 (PMC8164118; doi:10.2196/29014)
Supplement: Multimedia Appendix 2 [file resprot_v10i5e29014_app2.doc]

INFORMATION REQUEST

**Treatment efficacy:***PCORI is primarily interested in comparative clinical effectiveness research (CER), in which two or more interventions with robust prior evidence of efficacy are compared. Please address the existing evidence of efficacy for the specific version of computerize cognitive behavioral therapy (cCBT) being used in this study.  If insufficient data are available, please describe the specific differences between cCBT interventions with stronger evidence of efficacy and the program being proposed in the current study, and address why the current intervention represents an improvement over prior cCBT interventions (e.g., Web-MAP).  Similarly, please provide strong primary references to support the efficacy of pain education, and if available, references to support the specific version of pain education being used in this study.  If there are none available, please provide documentation to support the statement that pain education, in general, is "widely available" for patients with SCD (p. 1 of Research Plan).*

**Evidence on cCBT.** There is no evidence for the specific version of cCBT being tested in the CaRISMA project; however, with over 100 clinical trials testing different versions of technology-delivered CBT interventions, the efficacy of cCBT for mental health and pain and other somatic symptoms has been well established.^1^ In fact, a recent review shows that regardless of the cCBT version being tested, there is a moderate effect size for the impact of cCBT on pain disability and intensity, as well as positive spillover effects for mood outcomes.^2^ In addition to multiple systematic reviews showing the efficacy of cCBT on clinical outcomes, our co-Investigators have demonstrated that cCBT is effective for improving pain related disability in adolescence with pain.^3^ The type of online CBT program varies across the clinical trials referenced in the systematic reviews and the two trials completed by Palermo et al. However, the cognitive behavioral therapy content delivered is consistent across these cCBT programs and follows traditional CBT workbooks.

**How the current intervention represents an improvement over our prior cCBT interventions (e.g., Web-MAP).** The CaRISMA project cCBT program uses the same core content (CBT principles) as the Web-MAP and iCanCope programs. In the CaRISMA cCBT program, we have added an artificial intelligence component (i.e. a chatbot) to increase interaction and personalization, and we added more patient/family representation and stories throughout the course. Overall, we have worked to make the multimedia components of the cCBT program more engaging by adding new illustrations, video, background music, and sound effects. However, as with all technology the cCBT program can be updated. For instance, after expanding our testing group, we found out that not everyone liked the name of the program, “CALM SCD” and some felt the logo looked too much like it was from a pharmaceutical company. Changing the program name, logo, and theme is an example of a minor update that will improve uptake and engagement.

**Why we changed the existing cCBT programs when we already know they work.** In adult care, cCBT has been associated with poor completion rates.^4^ In our own data, we have found that African American primary care patients will complete less than half of the available cCBT sessions,^5^ and adults with SCD completed even less, an average of 3 sessions out of 8 available. ^6^ Despite these low engagement rates, patients still reported significant benefit. Given data suggesting the more sessions patients complete the more they improve ^7^, we see increase in engagement as critical to helping maximize the benefits of cognitive behavioral therapy on outcomes in SCD.

A qualitative study of cCBT users found that computerized therapy could be improved through personalizing and sensitizing content to individual users, recognizing the need for users to experience a sense of “self” in the treatment which is currently absent.^8^ As we move from efficacy trials to implementation and dissemination of this intervention approach, there is more of a need to leverage newer technologies to help fit varied settings and lifestyles and appeal to the widest range of patients.^9^ Artificial intelligence technology is one of those approaches.

**Evidence for the efficacy of pain education.** Overall, there is typically only a small to moderate effect of pain education or self-management education on chronic pain; however, the positive effect of education is consistent.^10-12^ Even when delivered via the Internet, education programs have been found helpful for improving pain outcomes.^13^ In children with SCD, we have evidence that an online sickle cell education program has benefit on perceived social support^14^ and in adults with SCD, pain management education is associate with fewer pain days.^15^ Unfortunately, the evidence for pain education in SCD is limited and the current pain education approaches lack the engagement and support structure of the educational programs offered via our community partners.

**Pain education is “widely available.”** Our community partners, e.g. Sickle Cell Warriors and SC101, are providing high-quality psychoeducation programs. A recent SC101 survey completed by 123 patients, family members and advocates found that “education” was the most accessed and beneficial content provided through SC101 website and social media outlets.

In addition to online access to education, almost all sickle cell clinics provide some form of education on self-management via a specific health educator or pamphlets that are handed out to families. Sickle Cell and Pain education is available freely through several websites including two partners we are currently working with or have worked with in the past, OneSCDvoice.com and PainAction.com

**Rationale for selection of intervention and comparator:** *There is concern regarding whether there is clinical equipoise between the two arms, as pain education/psychoeducation has been used as a control condition in prior CBT efficacy trials.  Please describe the process of selecting pain education as the most appropriate comparator to CBT, and why it was selected over other, more active comparators.  Please include other comparators considered in a tabular format. Also describe the significant "decisional dilemma" addressed by the current comparison for patients, providers, and other stakeholders (i.e., are patients and providers faced with a difficult choice between these two interventions?).*

**Why education is the primary comparator.** In addition to several studies showing its efficacy, our community partners on this project were the main driver for selecting pain education, or self-management education, as the primary comparator. All of our community partners, Sickle Cell 101, Sickle Cell Warriors, and Sickle Cell Community Consortium currently have education programs or have implemented some type of sickle cell education program in the past, thus, this was a topic area where they were comfortable and had experience they felt they could contribute to the project. However, the team did consider other potential non-pharmacological interventions and even considered a three-arm study. Our colleagues at Duke University published a review of all non-pharmacological interventions tested in SCD. The data from this review in combination with their consultation, input from our investigative team and other literature (e.g. ^16^), helped guide our selection of the intervention and decisions outlined below.

| Intervention considered | Evidence | Data in SCD | Notes |
| --- | --- | --- | --- |
| Problem Solving Skills Training Program (Bright IDEAS) | Strong evidence in oncology;  Supported by NCI | Yes – unpublished pilot data from Pitt | Stakeholders and the investigative team felt that PSST would be better served as a component of the CBT intervention than a stand-alone treatment. |
| Mindfulness based stress reduction (MBSR) | One SCD trial in progress of a telephonic mindfulness intervention^17^ | One SCD trial in progress of a telephonic mindfulness intervention^17^ | Some patients interviewed rejected the utility of mindfulness and meditation; may not be culturally appropriate for this group; community-based organizations had less experience with this type of intervention |
| Hypnosis | Only mixed evidence in other chronic pain conditions | Yes - Two published studies in SCD | The findings are mixed and not clear that the intervention can be accomplished remotely |
| Acceptance and commitment therapy | Growing evidence appears promising but still more robust trials needed | No | ACT appears to have potential, however, lack of experience among the investigative team ruled this treatment out. |
| Biofeedback therapy | Good evidence in other pain populations | Only one study in SCD but positive findings | Too expensive to implement in clinics |
| Social Support group | Good evidence in other populations | Yes - Some small studies showing a positive effect | No research on virtual (online) social support groups. Decided that online social support groups were not sufficient as a stand-alone intervention. This is already being provided through the CBOs so difficult to remove from one treatment group. Final decision was to include social support to both the education and CBT arm. |

**What is the “decisional dilemma”.** Patients and providers need to know whether accessing CBT is worth the effort. Barriers to CBT may not need be considered if psychoeducation and self-management training is sufficient to improve outcomes. Community based organizations know how to implement sickle cell and self-management education programs and have the experience doing this. Providing cognitive behavioral therapy often requires supervision of some type by a psychologist/psychiatrist and additional training for the staff, thus, adding another barrier or burden to implementation. In addition, there is some stigma to participating in CBT, as it is considered mental health treatment even if for pain only.

**Current clinical use of interventions***: Please clarify whether either intervention is currently in clinical use for chronic pain or SCD; if not, please describe the extent of prior use, even if pilot work, particularly for the pain education intervention.*

The cCBT app is currently being tested with adult patients at the University of Pittsburgh. The app content is based on prior cCBT programs tested in pediatric SCD and other pain populations, e.g. (^3, 18, 19^). The pain education intervention is based on content delivered via SC101 and Sickle Cell Warriors. This content is being delivered via social media and the organizations’ websites. We will, however, have to extract this content from its current format and modify it to be delivered through a chatbot app, similar to the cCBT app, to make an appropriate comparison.

**Industry partners:** *The role of industry partners Wondros and Robots & Pencils is unclear; specifically, the rationale for and extent of any planned adaptation requires clarification and a stronger rationale.  Please describe in greater detail why any adaptation is warranted, the types of adaptations planned, and how each industry partner will contribute to the study's implementation, with specific attention to whether any modifications made to the existing interventions are expected to increase usability, accessibility, or efficacy. Also, please address whether Wondros has any prior experience working with the proposed patient population.*

**Rationale for including industry partners and improving the current cCBT program.** We know that if patients and family members with SCD use the CaRISMA cCBT program, they will benefit. Thus, our ultimate goal is to see this program disseminated and used nationally, and eventually, internationally. We want the CaRISMA project to “go viral” on the Internet and social media space. To accomplish this, we need sophisticated, high-quality communications design and a strategy that will help our CaRISMA program and work for SCD stand out in a crowded and competitive landscape.

We have solicited the help of Robots & Pencils because they specialize in consumer facing apps and using artificial intelligence (AI) to engage customers. The CaRISMA project is larger than our internal team at the University of Pittsburgh is well-suited to manage and we are not on the cutting-edge of the most updated AI technology. There are chatbot features that Robots & Pencils can implement that will help us personalize the experience and better engage participants.

Our rational for including Wondros is essentially to make our program “cool”. The current multimedia content for the cCBT app was developed in an academic setting with some consultation from Wondros. Although our content is good, we can elevate the user experience with more hands-on industry perspectives and input. Wondros has expertise designing successful human-centered multimedia communications for business, philanthropy, public policy, and health care. Their work has had the ability to attract that type of public attention that we need for bringing awareness to the SCD community. One high visibility example of Wondros’ work is the 2008 “Yes We Can” video for the Obama campaign (<https://www.wondros.com/obama-campaign-2008/>) that generated well over 50 million views. Since this time, Wondros has moved their focus toward health care and has expressed strong interest in working with us on any one of our SCD projects. The proposed CaRISMA project fits perfectly with their expertise and public mission.

**Role of industry partners.** The cCBT program has two primary components that make it work: multimedia content (videos, audio clips, illustrations and photos) and an artificial intelligence-driven chatbot that pushes this content, in a personalized way, to the user.

Wondros will help our team elevate the quality of our current multimedia content. They will first work closely with our team and stakeholders during a formative phase of discovery that will ensure that the creative work is tailored and optimized for our target audience, adolescents and adults living with SCD. They will then work with us to revise the lesson 1 module for this project. We will follow them through the process of drafting content, storyboarding, engaging stakeholders to revise the designs and messaging, building the illustrations, on camera interviewing and video production work. By Wondros teaching and modeling this process, our team will then be able to replicate the process and produce a similar quality product for the other lessons and additional content we produce to teach and engage participants. Finally, Wondros will follow our team, community partners and stakeholders, throughout this project to create a short video documentary. This documentary will not only bring more awareness to the project and promote the education and cCBT program for SCD but it will also serve as a way to give back and acknowledge the contributions of everyone involved.

Robots & Pencils will primarily manage the chatbot feature for the project. The AI technology behind chatbots is rapidly advancing and these computer programs are better able to simulate real conversation and learn from the user than they were when we first started this work in 2016. Our current cCBT chatbot was developed by the research staff at the University of Pittsburgh in consultation with Robots & Pencils. To increase the scale and reach of our platform, the project would benefit from an industry partner who specializes in AI to manage the chatbot and add improved features to increase the personalized feel of the interactions. Working with Robots & Pencils, we will also have the ability to add more story lines of conversation content over the course of the study so that participants have more interactions with the chatbot. Our hope is that participants continue to use the chatbot and explore different content or revisit old content, even after they are finished all the lessons.

**Wondros’ experience working with patients living with SCD.** For Dr. Jonassaint’s NHLBI funded K-award focused on SCD, Wondros has served as an unpaid consultant contracted to teach him their human-centered qualitative research process. For the current proposal, we would like to extend this working relationship between Wondros and the University of Pittsburgh to the current CaRISMA project where Wondros will be able to work hands-on, with patients, alongside the research team. Although Wondros does not have direct experience with sickle cell patients face-to-face (e.g. live interviews), they have extensive experience with a broad range of patients in other disease types including cancer and Crohn’s disease. They led communications for the NIH's All of Us Research Program, as wells as the University of Pittsburgh CTSI, Pitt+Me research participation program, for which they designed the user experience and created all messaging, videos, and social media.

**Primary outcome:** *Per the funding announcement, PCORI encourages investigators to include well-validated outcome measures, with known psychometric properties.  The pain intensity outcome appears still to be in development.  Further, pain intensity is less frequently used as a primary outcome than functional impairment/disability in many studies of chronic pain populations.  Please consider whether the PROMIS pain measure could be moved from a secondary outcome to a primary or co-primary outcome.*

We would be willing to make the PROMIS Pain Interference a co-primary outcome. Pain was consistently rated as the #1 aspect of sickle cell that patients and family members wish was addressed. The reason for pain reduction often noted by our stakeholders was so that they could be more functional, “If I had less pain I could do more…” In addition, based on prior studies of cCBT, pain interference is likely to show a greater change than daily pain intensity. Thus, after discussion, the team does agree that PROMIS Pain Interference could very reasonably be considered a primary outcome.

**Recruitment plan:** *Please provide additional details to support the feasibility of the recruitment plan, as there is concern that the enrollment and retention rates (estimated attrition rate of 15%) in particular may be overly optimistic, particularly given the proposed population of underserved, minority patients with SCD. This may include providing details such as recruitment rate from prior, similar trials completed by the study team, or in progress at study sites. Please use these data to support the anticipated recruitment rate for the proposed study, including the anticipated number screened, eligible, enrolled, and retained for each study site.*

Each clinical site was included in this project because of consistently strong recruitment. At the University of Illinois at Chicago (UIC), the PCORI-supported PARTNER program has recruited 305 of 370 unique University of Illinois Hospital SCD inpatients (82.4%) into a longitudinal study for improving SCD care after discharge. In addition, UIC is part of the NIH U01 SCD Implementation Consortium that provides support for clinical trial infrastructure. The Duke University program, with over 900 active pediatric and adult patients, is also involved in the NHLBI U01 Dissemination and Implementation Consortium. Over the past 15 months, as part of this consortium, Duke has enrolled 291 adolescents and adults into the national registry, which includes surveys and medical record abstraction, with over 80% opting in for blood bio-banking.  Duke has been a high enrollment site nationally for several industry sponsored studies, including the recently completed Glycomimetic SCD study and ongoing Pfizer Rivipansel study. Further, Duke has a longstanding collaboration with the University of Pittsburgh focusing on mHealth research studies that have led to several publications.^20-27^ Vanderbilt University has had similar success with recent collaborative mHealth projects, seeing only 1 out of 10 patients not complete their follow-up survey in a cCBT for mental health pilot trial. ^6^

At Ohio State University, the SCD program has demonstrated it is able to recruit and retain patients by enrolling 284 adults with SCD for 13 clinical studies, 5 of which are ongoing. For the 8 studies that are completed, they have retained 151/152 (99%) of patients randomized. Similarly, the University of Pittsburgh has consistently been capable of high recruitment numbers driven by a large but cohesive research team and strong personal relationships with patients that come to clinic. For instance, the University of Pittsburgh’s large blood study cohort has enrolled 174 adult patients over the past 2 years. Only one patient has withdrawn from the study during that time. In addition, the Hydroxyurea Direct Observed Therapy study that required patients to take selfie videos while taking their medication retained 25/29 (86%) at 12 months.

In our team’s other study with Web-MAP in adolescents with SCD, participation rate was 60% for those families who could be reached; high levels of initial intervention engagement (>90%) and adherence (>70%) were demonstrated. Most participants (80%) completed posttreatment outcome and diary measures.^19^

Finally, our CBO partners have been successfully encouraging patients to enroll into clinical trials. The current study will be a new role for the CBOs, where they will be providing the actual link for patients to enroll in this trial remotely. However, the CBOs investigators do have experience administering online questionnaires to their networks with success. Within two weeks, Sickle Cell Warriors was able to have over 200 patients complete an online survey, and SC101 had 123 participants complete an online survey in less than 10 days. We expect that engagement online and through social media via our community partners could potential be even more fruitful than engagement through the academic clinics.

**This study leverages technology and engagement to prevent attrition**. Our planned attrition rate is based on the number of patients we expect to complete at least one follow-up assessment. Typical dropout rates for adult SCD studies are as high as 50%. We have chosen clinical sites and CBOs that have demonstrated much better retention. Also, studies that have high attrition tend to require patients to return to clinic to complete clinical assessments. In the proposed study, we have the advantage of remotely accessing patients and administering assessments via their mobile device. These assessment tools are the exact electronic patient reported outcomes measures administered in clinic. Patients strongly prefer this approach the electronic survey approach over phone assessments. Finally, we are providing an intervention that will allow us to “push” new, tailored content to participants over the course of the study as brief intervention boosters. Not only will participants be able to continue having virtual conversations and explore content through the chatbot but we are keeping our participants engaged throughout the duration of the study with their continued expectation of the next exciting or interesting thing to read, see or hear.

**Opioid use:** *The application lacks details about the role of opioids in managing SCD pain. Given the common use of opioid medications in patients with SCD, discuss whether additional opioid-related outcomes of interest could be examined, such as average morphine equivalent dose (MED).*

We agree on the importance of opioid use in SCD and decreasing opioid use is a primary objective we have highlighted with our patient partners. However, measuring opioid use is challenging in any population, SCD is no exception. There have been very few studies to document opioid use in SCD. This has the potential to be the largest study to report opioid use in an adult SCD population.

**More details on methods for opioid use assessment in the proposed project.** All patients in the study would be asked to enter electronically their prescriptions, dosing and when they take their medication. We will ask daily (“did you take your [opioid] medication today”) and weekly (“how many times have you taken your [opioid] medication this week?”). For the clinical patients, we will be able to confirm number of scripts written via PCORnet data. We are open to exploring contacting pharmacies for each patient. In this scenario, with written consent and a signed release of information from participants, a study staff member would contact the participants’ pharmacy (or pharmacies) at the12- month assessment to verify the self-reported information provided by the participants in the prior assessments.

For the opioids dosing, we are following the methods used in previous study on prescription records for 203 adults with SCD.^28^ In brief, the oral morphine equivalents or morphine equivalent dose ratios would be as follows: 1 mg codeine = 0.13 mg OME, 1 mg hydrocodone = 1 mg OME, 1 mg hydromorphone = 5 mg OME, 1 mg methadone = 4.7 mg OME, 1 mg tramadol = 0.2 mg OME, 1 mg oxycodone = 1.5 mg OME, 1 mg tapentadol = 0.4 mg OME, and transdermal fentanyl is converted based on 1 mcg/hr = 3 mg OME/daily.

**Evaluating opioid risk, misuse and dependence.** Other studies have recommended opioid screening tools for assessing the connection between pain and opioid treatment. This approach would allow us to assess between treatment group differences in opioid misuse risk. A table of suggested opioid screening tools that could potentially be added to the project are below. ^29^

| Measure | | Notes |
| --- | --- | --- |
| Opioid Risk Tool | ORT | Brief self-assessment for patients with chronic pain being considered for opioid therapy. Tested on new patients treated in a pain clinic. Ten weighted risk items. Good sensitivity and specificity. |
| Screener and Opioid Assessment for Patients with Pain—Revised | SOAPP-R | Revised, shorter version of original (decreased from 142 to 24 items.) Intended for use with persons with chronic pain. Screens risk for aberrant medication-related behavior. Good reliability and validity. |
| Screening Instrument for Substance Abuse Potential. | SISAP | Assesses risk of opioid dependency in those with substance abuse history. Contains five items. Good sensitivity and specificity. Used by primary care providers. Not validated by pain patients. |
| Current Opioid Misuse Measure | COMM | For pain patients already on long-term opioid therapy. Used to assess adherence to opioid prescribing. Tested on 277 patients with chronic noncancer pain. 17 items. Adequately measures aberrant behavior, excellent internal consistency, and test-retest reliability |

REFERENCES

**1.** Andersson G. Internet-Delivered Psychological Treatments. *Annu Rev Clin Psychol.* 2016;12:157-179.

**2.** Buhrman M, Gordh T, Andersson G. Internet interventions for chronic pain including headache: A systematic review. *Internet Interv.* 2016;4:17-34.

**3.** Palermo TM, Law EF, Fales J, Bromberg MH, Jessen-Fiddick T, Tai G. Internet-delivered cognitive-behavioral treatment for adolescents with chronic pain and their parents: a randomized controlled multicenter trial. *Pain.* 2016;157(1):174-185.

**4.** Knowles SE, Lovell K, Bower P, Gilbody S, Littlewood E, Lester H. Patient experience of computerised therapy for depression in primary care. *BMJ Open.* 2015;5(11):e008581.

**5.** Jonassaint CR, Gibbs P, Belnap BH, Karp JF, Abebe KK, Rollman BL. Engagement and outcomes for a computerised cognitive-behavioural therapy intervention for anxiety and depression in African Americans. *BJPsych Open.* 2017;3(1):1-5.

**6.** Jonassaint C, Kang C, Prussien K, et al. Feasibility of Implementing Mobile Technology-Delivered Mental Health Treatment in Routine Adult Sickle Cell Disease Care. *Translational Behavioral Medicine.* In press.

**7.** Rollman BL, Herbeck Belnap B, Abebe KZ, et al. Effectiveness of Online Collaborative Care for Treating Mood and Anxiety Disorders in Primary Care: A Randomized Clinical Trial. *JAMA Psychiatry.* 2018;75(1):56-64.

**8.** Knowles SE, Toms G, Sanders C, et al. Qualitative meta-synthesis of user experience of computerised therapy for depression and anxiety. *PLoS One.* 2014;9(1):e84323.

**9.** Andersson G, Titov N, Dear BF, Rozental A, Carlbring P. Internet-delivered psychological treatments: from innovation to implementation. *World Psychiatry.* 2019;18(1):20-28.

**10.** Du S, Yuan C, Xiao X, Chu J, Qiu Y, Qian H. Self-management programs for chronic musculoskeletal pain conditions: a systematic review and meta-analysis. *Patient Educ Couns.* 2011;85(3):e299-310.

**11.** Warsi A, Wang PS, LaValley MP, Avorn J, Solomon DH. Self-management education programs in chronic disease: a systematic review and methodological critique of the literature. *Arch Intern Med.* 2004;164(15):1641-1649.

**12.** Mehlsen M, Heegaard L, Frostholm L. A prospective evaluation of the Chronic Pain Self-Management Programme in a Danish population of chronic pain patients. *Patient Educ Couns.* 2015;98(5):677-680.

**13.** Ruehlman LS, Karoly P, Enders C. A randomized controlled evaluation of an online chronic pain self management program. *Pain.* 2012;153(2):319-330.

**14.** Hazzard A, Celano M, Collins MH. Effects of STARBRIGHT World on knoweldge social support and coping in hospitalized children with sickle cell disease and asthma. *Children’s Health Care.* 2002;31(1):69-86.

**15.** Gil KM, Carson JW, Sedway JA, Porter LS, Schaeffer JJ, Orringer E. Follow-up of coping skills training in adults with sickle cell disease: analysis of daily pain and coping practice diaries. *Health Psychol.* 2000;19(1):85-90.

**16.** Anie KA, Green J. Psychological therapies for sickle cell disease and pain. *Cochrane Database Syst Rev.* 2015;5:CD001916.

**17.** Williams H, Silva S, Simmons LA, Tanabe P. A telephonic mindfulness-based intervention for persons with sickle cell disease: study protocol for a randomized controlled trial. *Trials.* 2017;18(1):218.

**18.** Palermo TM. *Cognitive-behavioral therapy for chronic pain in children and adolescents*. 1 ed. Oxford;New York;: Oxford University Press; 2012.

**19.** Palermo TM, Dudeney J, Santanelli JP, Carletti A, Zempsky WT. Feasibility and Acceptability of Internet-delivered Cognitive Behavioral Therapy for Chronic Pain in Adolescents With Sickle Cell Disease and Their Parents. *J Pediatr Hematol Oncol.* 2018;40(2):122-127.

**20.** Jonassaint CR, Kang C, Prussien KV, et al. Feasibility of implementing mobile technology-delivered mental health treatment in routine adult sickle cell disease care. *Transl Behav Med.* 2018.

**21.** Vaughn J, Jonassaint J, Summers-Goeckerman E, Shaw RJ, Shah N. Customization of the TRU-PBMT App (Technology Recordings to better Understand Pediatric Blood and Marrow Transplant). *J Pediatr Nurs.* 2018;42:86-91.

**22.** Jonassaint CR, Rao N, Sciuto A, et al. Abstract Animations for the Communication and Assessment of Pain in Adults: Cross-Sectional Feasibility Study. *Journal of medical Internet research.* 2018;20(8):e10056.

**23.** Anderson LM, Leonard S, Jonassaint J, Lunyera J, Bonner M, Shah N. Mobile health intervention for youth with sickle cell disease: Impact on adherence, disease knowledge, and quality of life. *Pediatric blood & cancer.* 2018;65(8):e27081.

**24.** Jonassaint CR, Kang C, Abrams DM, et al. Understanding patterns and correlates of daily pain using the Sickle cell disease Mobile Application to Record Symptoms via Technology (SMART). *British journal of haematology.* 2018;183(2):306-308.

**25.** Leonard S, Anderson LM, Jonassaint J, Jonassaint C, Shah N. Utilizing a Novel Mobile Health "Selfie" Application to Improve Compliance to Iron Chelation in Pediatric Patients Receiving Chronic Transfusions. *J Pediatr Hematol Oncol.* 2017;39(3):223-229.

**26.** Jonassaint CR, Shah N, Jonassaint J, De Castro L. Usability and Feasibility of an mHealth Intervention for Monitoring and Managing Pain Symptoms in Sickle Cell Disease: The Sickle Cell Disease Mobile Application to Record Symptoms via Technology (SMART). *Hemoglobin.* 2015;39(3):162-168.

**27.** Shah N, Jonassaint J, De Castro L. Patients welcome the Sickle Cell Disease Mobile Application to Record Symptoms via Technology (SMART). *Hemoglobin.* 2014;38(2):99-103.

**28.** Han J, Saraf SL, Zhang X, et al. Patterns of opioid use in sickle cell disease. *Am J Hematol.* 2016;91(11):1102-1106.

**29.** Puntillo K, Naidu RK. Measurement of Chronic Pain and Opioid Use Evaluation in Community-Based Persons with Serious Illnesses. *Journal of palliative medicine.* 2018;21(S2):S43-S51.
